# Machine learning reveals the waggle drift’s role in the honey bee dance communication system

**DOI:** 10.1093/pnasnexus/pgad275

**Published:** 2023-08-25

**Authors:** David M Dormagen, Benjamin Wild, Fernando Wario, Tim Landgraf

**Affiliations:** Department of Mathematics and Computer Science, Freie Universität Berlin, 14195 Berlin, Germany; Department of Mathematics and Computer Science, Freie Universität Berlin, 14195 Berlin, Germany; Department of Electronics, Universidad de Guadalajara, Guadalajara, 44430 Jalisco, Mexico; Department of Mathematics and Computer Science, Freie Universität Berlin, 14195 Berlin, Germany

**Keywords:** animal behavior, entomology, *Apis mellifera*, machine learning, neural networks

## Abstract

The honey bee waggle dance is one of the most prominent examples of abstract communication among animals: successful foragers convey new resource locations to interested followers via characteristic “dance” movements in the nest, where dances advertise different locations on different overlapping subregions of the “dance floor.” To this day, this spatial separation has not been described in detail, and it remains unknown how it affects the dance communication. Here, we evaluate long-term recordings of *Apis mellifera* foraging at natural and artificial food sites. Using machine learning, we detect and decode waggle dances, and we individually identify and track dancers and dance followers in the hive and at artificial feeders. We record more than a hundred thousand waggle phases, and thousands of dances and dance-following interactions to quantitatively describe the spatial separation of dances on the dance floor. We find that the separation of dancers increases throughout a dance and present a motion model based on a positional drift of the dancer between subsequent waggle phases that fits our observations. We show that this separation affects follower bees as well and results in them more likely following subsequent dances to similar food source locations, constituting a positive feedback loop. Our work provides evidence that the positional drift between subsequent waggle phases modulates the information that is available to dance followers, leading to an emergent optimization of the waggle dance communication system.

**Significance Statement**

The honey bee waggle dance is a rare example of abstract communication. Using stereotypical body movements, bees advertise resource locations on the “dance floor” in the hive. While the dance message itself is well understood, the structure of the emergent dance floor is largely unknown. Here, we combine long-term data acquisition and machine learning to showcase a previously neglected property of the dance motion. We show that dancers continuously drift along their waggle direction, pulling dance followers outwards on the dance floor. This simple bias at the individual level compartmentalizes the available space into subregions that spatially represent different sectors of the environment. This likely improves the efficacy of their communication system and demonstrates the elegance of emergence in a complex system.

## Introduction

**Fig. 1.**
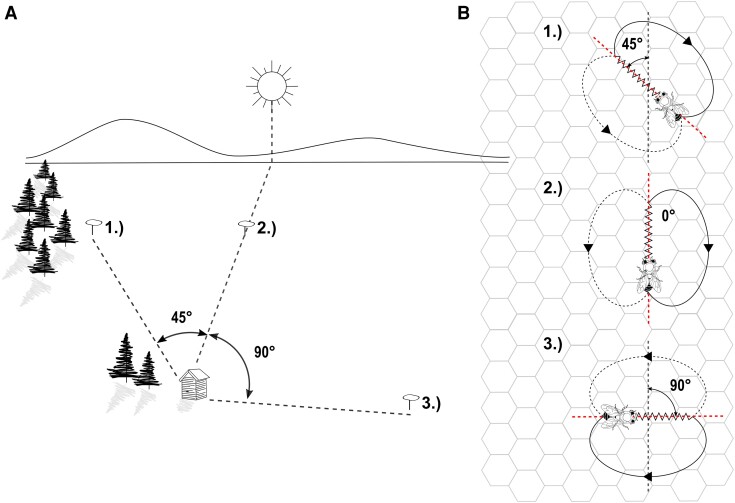
Correlation between waggle dance properties and food locations in the field. A) three food sources in the field located at 1) 45° counterclockwise 2) 0° and 3) 90° clockwise, with respect to the sun's azimuth. B) Their representation through waggle dance paths on the surface of a vertical honeycomb. The figure is available under CC BY 4.0 and was adapted from Wario et al. (7).

Honeybees use an elaborate communication system to communicate newly found food sources to other workers (1). In the so-called “waggle dance,” foragers that return from a valuable feeding site move in a repeated, stereotypical pattern over the comb surface followed by the so-called dance followers, bees that move in an equally stereotypical pattern who may be recruited to the new food source by doing so. The dancer first performs fast lateral body oscillations while moving forward on an approximately straight line, then turning alternatingly into left and right turns that bring her back to the start of her previous “waggle phase.” Dances may consist of dozens of waggle and return phases, and the duration a dancer engages in dance communication has been shown to correlate with the food profitability (2, 3). Intriguingly, the dance contains more information than simply that a food source exists somewhere outside. To the human observer, it conveys a distance and a direction, and dance recruits have been shown to use the vector information contained in the dance (1, 4–6). Postulated already in the 1940s by Karl von Frisch, the direction to the food source is reflected in the dancer’s body orientation with respect to the vertical during the waggle phase (1, 4, 5). The reference frames (zero degrees) are the sun’s azimuth in the field, and upwards in the hive. Hence, the forager’s dance pattern is rotated to correct for the sun’s motion through the sky over the day. The distance to the feeder is encoded by the waggle phase’s duration (see Fig. 1 for a visual explanation of the dance).

The dance followers enact a similar periodic motion, touching the dancer with their antennae alternatingly from both sides, running after her in the return phases. In the darkness of the hive, dance followers have to integrate a variety of cues to understand their own orientation with respect to that of the dancer all the while measuring the duration of the dancer’s body oscillation, which could be as short as a few hundred milliseconds (8, 9). Since the relevant properties all relate to the waggle phase, it can be thought of as an information packet, while the return phase serves to localize the dancer to an approximate spot on the comb during the dance.

**Fig. 2.**
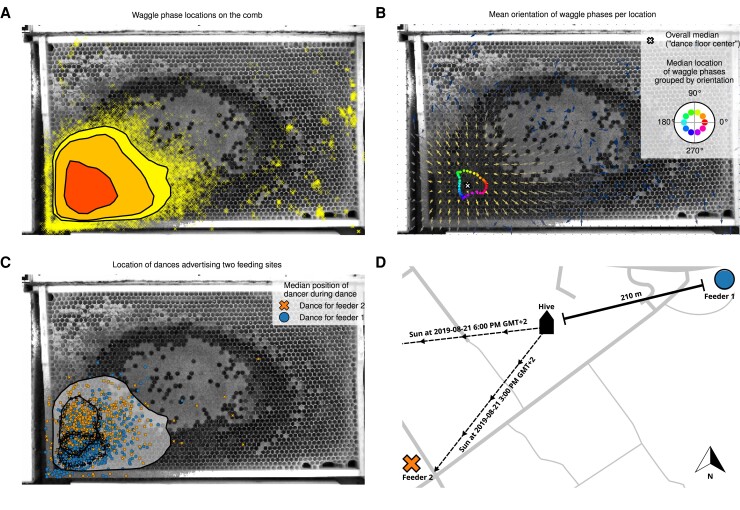
Automated detections of waggle phases. A) Each automatically detected waggle phase (N=84,628) over 32 days is displayed on top of a background-subtracted image of the left side of our observation hive from 2019. As the scatterplot is too dense in the central region, a contour plot indicates 50, 90, 95% of the density. Most of the waggle phases are located in a small area between the hive entrance (bottom left) and the brood nest (center). B) Mean waggle orientation over location. Calculating the mean waggle orientation for a $1\;{\mathrm{cm}}^{2}$ grid with all detected waggle phases of the Berlin 2019 season (N=84,628) shows a clear outward pointing pattern of directions. The mean was calculated over the normalized direction vectors in each bin, thus the arrow length for bins with an even distribution of directions is short and the length for bins with a narrower distribution is longer. The arrows are colored according to the same density distribution as in subplot *a* with arrows in the dense region in bright yellow and arrows with only few data points in dark blue. The scatterplot in the center of the dance floor shows the median location of all waggle phases grouped by their orientation in 5${}^{\circ }$ steps. E.g. the median location of waggle phases that point approximately to the right (0${}^{\circ }$) is located to the right of the dance floor center. C) The scatterplot shows the median position of each automatically recognized dance from bees returning from feeder 1 (blue circles) or feeder 2 (orange crosses) (N=1,497). The two overlapping contours give the 50% density area for feeder 1 (dashed) and feeder 2 (solid). The large area shows the 95% data interval of all waggle phases again for reference. Dances advertising the different feeders were performed in different regions on the dancefloor, albeit with a large overlap and a high variance of the dance locations. D) Schematic of the feeder experiment conducted in 2019. One of the equidistant feeders was located approximately towards the sun and the other away from the sun. As we recorded most dances between 3 PM and 6 PM (Greenwich Mean Time (GMT)+2), we show the corresponding solar azimuths at the beginning of the experimental period (2019 August 21). Until the end of the period (2019 September 15), the cone became slightly narrower as the sun changed its path.

Bees organize the colony space into different functional compartments (10–12) and communication dances are performed predominantly on a region close to the entrance, called the “dance floor” (1, 13–15) (see also Fig. 2). This dance floor is not a homogenous area where a returning forager will perform her dance in a random location, however. Boch (16) first described a separation of dancing foragers based on the distance to the advertised food source and the direction of the waggle phase: bees advertising farther food sources would also dance at a location farther away from the hive entrance compared to foragers advertising closer resources and the dance floor of both groups would change as the direction of the waggle phase changed with the sun over a day. Seeley et al. (17) described a separation based on the compass direction to the foraging site and thus based on the direction of the waggle phase, which was attributed to a drift between subsequent waggle phases: throughout her dance, a dancer’s return phase would not lead her back to the exact same spot where the last waggle phase started but to a position slightly offset in the direction of the waggle phase. The different waggle phases of a dance are therefore not localized to a confined spot on the dance floor but are performed in multiple locations over a dance. It has been suggested that this might be an adaptation to help a dancing bee “broadcast her dance information over much of the dance floor” (6). Landgraf et al. (18) first measured the magnitude of the forward and sideward drift between subsequent waggle phases for one food source at a distance of 230 m.

While the spatial separation has been mentioned in the literature, the effects on the follower bees are unknown: do follower bees that follow different dances separate on the dance floor as well? Does the position of a follower bee already predict the dances and advertised feeding sites she can observe? Does this subdivision of the dance floor allow dancers and dance followers to actively target a certain audience? We used long-term recordings to identify and track individual bees with the BeesBook system (19–21), 2D-convolutional neural networks to detect and decode individual waggle phases from an additional video stream, 1D-convolutional neural networks to classify dance and following behavior from the spatiotemporal tracking data, and sucrose feeders equipped with cameras to detect and identify the foraging bees in the field. Using modern machine learning techniques, we created an unprecedented dataset to describe the waggle drift, confirmed our observations with a simple mathematical model, and investigated how the separation of dances influences the follower bees. We show that the location of individual waggle phases on the comb is correlated to the body orientation of the dancer during the waggle phase. Moreover, we observed that dances advertising different feeding sites tend to occupy different regions of the dance floor and that the distance between the different dancers as well as the distance between the follower groups increased over the course of a dance. We find that the position where a bee starts following a dance is strongly correlated to the direction advertised by the dance. We then specifically looked at situations where a bee followed an initial dance to either feeder and after this interaction, there were at least two other dances to the two different feeders available. In that situation, the follower bee was more likely to follow the closer dance and dances advertising the same feeder as the initial dance were on average closer.

## Results

In our one-frame observation hive we detected waggle phases automatically using software described previously (7), which we modified to further improve detection results (see Materials and methods section for details).

In 2019, we detected 84,628 (14,404) waggle phases on the left (right) side of the comb on 32 (31) days. In 2021, we detected 36,367 (6,390) waggle phases on the left (right) side of the comb on 47 (33) days. The waggle phases always clustered strongly in a region around the hive exit and we recorded more dance activity on the left side of the comb in both seasons, see Fig. 2A and online supplementary Fig. S1 for both datasets and sides. We calculated the mean waggle orientation for all waggle phases detected inside each $1\;{\mathrm{cm}}^{2}$ bin on our observation comb. These mean orientations appeared to point outwards from the center of the dance floor, see Fig. 2B. To confirm the correlation between the location where a waggle phase is performed on the comb and its orientation, we calculated the offset vector pointing from the dance floor center (the overall median waggle phase location) to the location of each individual waggle phase. We then calculated the dot product between this normalized offset vector and the waggle phase orientation. This value ranges between −1 (if the two orientations are opposite from each other) and 1 (if the two orientations align). We found that the median dot product is significantly greater than 0 in all datasets (P<0.05 in the dataset from the right side in Berlin 2021, P<0.0001 in the other three datasets; one-sided sign test; for median values, test statistics, and histograms see online supplementary Fig. S2). Therefore, we confirmed that individual waggle phases are on average shifted in the direction the dancer faced throughout the waggle phase. In 2019, we trained two groups of bees to two equidistant feeders offering highly concentrated sucrose solution. Both feeders were located ca. 210 m away from the hive, with feeder 2 approximately in the direction of the sun during our experimental hours and feeder 1 in the opposite direction (see Fig. 2D for a schematic and Materials and methods section for details). Visits to each feeder were video-recorded by a dedicated camera and the ID of the forager was obtained automatically using the BeesBook system (see Materials and methods section). The location of the dances performed by these animals after returning to the hive was automatically extracted (N=1,993 total, N=1,497 left side of the comb, N=496 right side). While the feeders were mostly opened around the early afternoon, the exact times varied over the experimental days. We recorded 79.2% of the dances between 3 PM and 6 PM (GMT+2). Additionally, the solar azimuth during those times changed slightly over the season, see Fig. 2D for two representative sun directions. Both groups thus comprised dances with slightly varying expected waggle phase orientations, yet dances advertising feeder 2 were always roughly oriented upwards in the hive with dances to feeder 1 being performed in the opposite orientation. We found that dances advertising these feeders each covered a similar area as the overall dancefloor (see Materials and methods section for details and online supplementary Table S1 for an overview of the data used). On the left side, the overall dancefloor estimated from all detected waggle phases covered 11.64%±1.86% (N=16 days; mean±std) of the comb with dances to feeder 1 covering 9.46%±2.41% (N=8 days) and dances to feeder 2 covering 10.20%±3.43% (N=8 days). On the right side, the overall dancefloor covered 7.10%±1.80% (N=16 days) of the comb with dances to feeder 1 covering 7.89%±1.99% (N=7 days) and dances to feeder 2 covering 9.50%±2.61% (N=6 days). However, we found that the regions covering 50% of the dances of the respective feeders and thus their median positions were significantly different from each other (left side: P<0.0001, $\chi^{2}=236.26$, N=1,496; right side: P<0.0001, $\chi^{2}=43.1$, N=496; Mood’s median test on the median vertical positions), see Fig. 2C for the left side of the comb and online supplementary Fig. S3 for the right side. This displacement corresponded to the expected orientation of the respective waggle phases, with the dances advertising feeder 2 roughly oriented upwards in the hive. Even though the expected orientation of the individual dances changed over the time of the day and the season, a clear separation is visible even when all data are shown together.

**Fig. 3.**
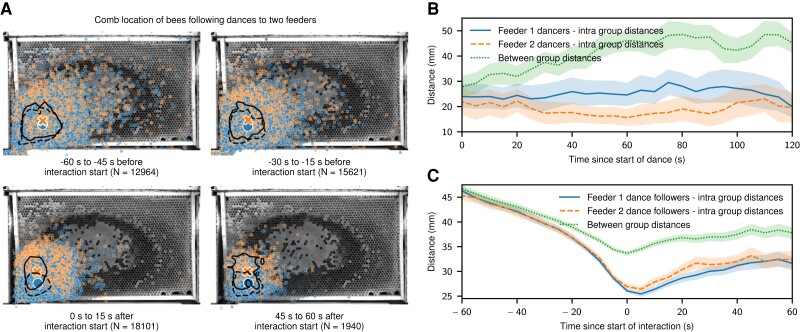
Locations and group distances of dancers and followers. A) Median locations of follower bees in a specific time window during a dance-following interaction. Each dot is the location of a dance follower following a dance to feeder 2 (orange crosses) or feeder 1 (blue circles). The large markers indicate the overall group means (orange cross: feeder 2, blue circle: feeder 1). The contours show the 50% density area for feeder 1 (dashed) and feeder 2 (solid). B) Mean intra- and inter-group distances for bees dancing either towards feeder 1 or feeder 2 over the course of the dance (N=55 dances). The bands indicate a 95% CI of the mean. The distance between dancers for the two different feeders increases over the course of a dance. C) Mean intra- and inter-group distances of bees following dances to the two feeders around the start of a dance-following interaction (N=18,101 dance-following interactions). The bands indicate a 95% CI of the mean. While there is no difference between the groups more than ca. 30 s before the beginning of the interaction, it becomes apparent as the interaction starts.

**Fig. 4.**
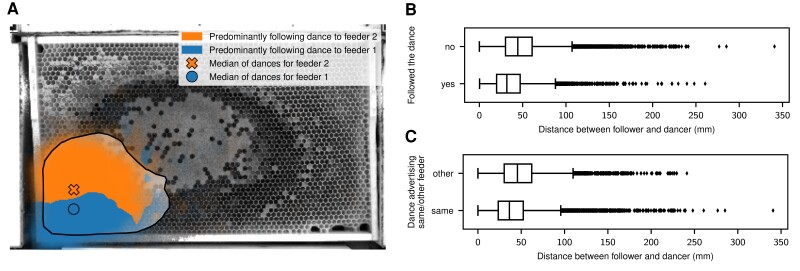
Effect on dance-following interactions. A) For each dance-following interaction, the initial positions of the followers (N=24,777) were collected into a $1\;{\mathrm{cm}}^{2}$ grid and for each of these bins the most-frequent dance target was calculated. The area inside 90% of the density of data points is opaque, with the remaining bins being transparent. The two markers indicate the overall median of the median positions of dancing bees. In the background, the same 95% data interval from Fig. 2A is shown for reference. B) The distance between the follower bee and a candidate dancer for all situations where a follower bee had subsequent dances for both feeders available after her initial dance-following. The distance to the dance(s) that the bee decided to follow was on average lower than other dances the bee did not follow. See online supplementary Fig. S7 for a histogram of the data. C) Same data as in B but grouped by whether the subsequent dance advertised the same or the other feeder. Subsequent dances by other individuals advertising the same feeder were on average closer to a follower bee than dances advertising the other feeder. See online supplementary Fig. S8 for a histogram of the data.

As suggested previously (6, 17), we hypothesized that the separation of dances that advertise different food source locations and the drift throughout a dance arise naturally from the properties of the waggle dance. It was shown before that a dancer does not fully return to her original location on the comb between waggle phases but instead has a slightly positive drift towards the direction indicated by the waggle (16–18). We confirmed this by looking at all subsequent waggle phases of our manually annotated ground truth data (N=820 pairs of waggle phases) and calculating the distance of the start of each waggle phase with respect to the start of the previous phase, projected on the waggle direction with the dot product. We found a drift with a high variance but a positive mean (0.81±7.3mm), see the histogram in online supplementary Fig. S6. We used the measured values to parameterize a simple motion model and simulated 2,000 dances with a random start location normally distributed around a comb location and each with a random waggle direction (see Materials and methods section for details). For each dance, we simulated 20 waggle phases. We found that this model based solely on the drift between subsequent waggle phases yields a similar pattern of mean waggle orientation per spatial bin and a positive median of the dot product between waggle orientations and dance floor center as observed in our datasets (median=0.325, P<0.0001, one-sided sign test with 23,695 of 40,000 samples larger than 0), see quiver plot and histogram in online supplementary Figs. S4 and S5. For a sample of 55 dances, we annotated the time of the first waggle phase down to a precision of approximately 166 ms using our video recordings. For those, we calculated the intra-group and between-group distances for the two feeder groups and confirmed that the distance between the dancers of different groups is consistently higher than between dancers within the same group (P<0.0001, $\chi^{2}=52.51$, N=110, Mood’s median test on the median intra- and inter-group distances for each dance) and increases during the dances, see Fig. 3B. We confirmed the same effect for the dance followers by extracting the position of the follower bees before and during each dance-following interaction. As with the dancing bees, we saw a clear separation during the dance as well as slightly before the start of the dance (P<0.0001, $\chi^{2}=269.41$, N=27,300, Mood’s median test on the median intra- and inter-group distances for each follower). This separation developed ca. 30 s before the dance started, see Fig. 3. To quantify whether there was a difference in the information a candidate follower was exposed to depending on her location on the comb, we grouped the initial position of the follower bee for all dance-following interactions for each $1\;{\mathrm{cm}}^{2}$ bin and calculated the most-frequent dance target for that bin. We saw a clear boundary where bees that started following a dance above that boundary were more likely to follow a dance to feeder 2 and vice versa below the boundary, see Fig. 4A. We looked at all interactions where after an initial dance-following event, at least two dances from another two individuals advertising the two different feeders were available and the initial follower subsequently followed any of these. We recorded this situation 37,774 times from 672 dances performed by 100 unique dancers on 12 unique days with 925 unique followers. We found that the distance from the follower to the subsequently followed dance (35.71±22.24mm; mean±std) was significantly lower than to the dance that was subsequently ignored (48.23±24.23mm; mean±std; Mood’s median test: P<0.0001, $\chi^{2}=1,677.63$), see Fig. 4B. Disregarding which subsequent dance was followed, we found that the distance to the dances advertising the same feeder (39.54±23.75; mean±std) was significantly lower than to the dances advertising the other feeder (47.05±24.07; mean±std; Mood’s median test: P<0.0001; $\chi^{2}=824.35$), see Fig. 4C.

## Conclusion

Using long-term recordings of honey bees in combination with several machine learning models and different data sources, we created a dataset of unprecedented scale to describe a property of the honey bee waggle dance: dances to different food sources tend to separate on the dance floor throughout a dance. We automatically recognized and decoded waggle phases in our observation colonies from a high-framerate video stream, and additionally tracked and identified all individuals in the hive from a lower framerate, high-resolution recording. Additionally, we ran a feeder experiment over several weeks, recording and identifying the foraging individuals using cameras mounted at the feeders. We used machine learning to recognize dancing and dance-following bees inside the hive. In both years we observed more dance activity on the left side of the comb, which we had also already observed in a previous year (22). This may be caused by a slight bend in the exit tube which leads to returning foragers primarily moving onto the left side. However, we do not think this affects our results. We confirmed the previously described property of waggle dances, a forward drift between successive waggle phases within a dance, that leads to a separation of dances advertising different feeding sites and showed that a simple motion model can explain our observations. We then looked at how this drift affects the dance followers. We showed that the location where a bee starts following a dance is correlated with the advertised food source location. We also showed that, if a follower bee has at least two dances to different feeding sites available after following an initial dance from a third individual, she will (1) more likely follow the closer dance and (2) a dance to the same feeder will on average be closer. Our findings indicate that the dance floor area in the hive is not homogenous, but instead emerges as the aggregation of subregions that represent different sectors of the environment. While our experimental conditions slightly vary over the recording period and between the years (specifically, we use both scented and unscented sucrose solutions of varying concentrations), we believe that this does not affect our findings. Still, it would be interesting to study the effect of different conditions in isolation of each other such as the number of feeders, the distance to the feeders, and the odor and concentration of the offered sucrose solution on the waggle drift and the emergent dance floor.

We hypothesize that the waggle drift, as a simple mechanical property of the waggle dance, might help the dancing bee to match the information transmitted via the dance better to a specific audience. We know that bees do not compare dances advertising different feeding sites before flying out (14). Instead, a follower bee that is more likely to see and follow successive dances to a similar location might be more likely to fly out and find the feeding site, as the number of dances a bee follows before flying out increases her chance of finding the advertised location (23). A follower bee might follow dozens of individual waggle phases before being successfully recruited (reviewed in Ref. (24)), and following more waggle phases increases her success at finding a food site (25–27). Thus, by leading the follower bees to a specific region on the dance floor where they are more likely to observe similar dances, the waggle drift might increase their chance to be successfully recruited, constituting a positive feedback loop. Additionally, by increasing the probability that a follower observes successive dances by different individuals that advertise similar locations, the waggle drift might increase a follower’s decoding accuracy, as there is a substantial error in the communicated information which may be averaged out by following more waggle phases (18, 28–31). We also know that dance-following often does not convey new spatial information but instead reactivates already proficient foragers (24, 32, 33) and that idle foragers sit at the outer boundary of the dance floor but can still be activated to forage if they encounter a dance (13). It would be an interesting venue for future research to check whether idle foragers optimize their location on the comb to observe dances pointing to specific directions to only be activated by highly motivated and thus, through an increase in waggle-bouts (3, 34) likely further drifting, dancers. Experiments with a biomimetic waggle dance robot (35) could show whether dances at a location that matches the drift expected from the waggle direction attract more followers and are thus more successful.

## Materials and methods

### Colony setup

#### Berlin 2019

We set up a queenright colony with approximately 1,500 bees in a one-frame observation hive (located at 52.457130, 13.296285) on 2019 July 4. We incubated brood from a sister colony in an external incubator. Every day, all hatched bees in the incubator were removed and manually marked with circular, curved tags. The last batch of bees was added on 2019 October 15. We exchanged the comb in the colony approximately every 21 days to prevent unmarked bees from emerging inside the colony. We glued four fiducial markers to the edges of the hive just outside of the comb to calculate a homography that allows transforming image coordinates into a common reference frame. The colony was connected to the outside via a plastic tube. The tube ended in a box where we installed a Raspberry Pi camera 2 to record incoming and outgoing bees at 10 frames per second.

#### Berlin 2021

We set up a colony at the same location and observation hive as in 2019 with ca. 2,000 unmarked bees in the beginning of August. No bees were marked and young bees were allowed to emerge from the brood nest inside the hive. We recorded the dance activity with our waggle dance recognition software between 2021 August 12 and 2021 September 30.

### BeesBook tracking system

Bees marked with a tag could be identified and localized using an automated observation system called “BeesBook” (19–21). To create suitable data for the BeesBook system, an observation hive is placed into a camera rig that allows filming high-resolution images at 6 Hz over extended periods. The hive is lighted with infrared flashes synchronized to the exposure timings of the cameras, both sides are alternatingly imaged to avoid back-lighting. The image recordings are encoded into short video files and uploaded to a server for later analysis. A pipeline of software components then processes the data which yields IDs, positions, and orientations for all marked animals. The data are transferred to a database for optimized access in the analyses conducted.

### Training of foragers to artificial food sources

#### Berlin 2019

From 2019 August 21 to 2019 September 15, we ran an experiment with sucrose feeders that were equipped with cameras to record feeding bees. The 3D printed feeders (made from polylactic acid) were equipped with a RaspberryPi and a Raspberry Pi camera 2 running at 10 frames per second. The cameras saved short video snippets when any motion was detected using background subtraction. The software used to record the images was the same for the entrance camera and the feeder cameras. It is available on GitHub.^1^ Starting on 2019 August 8, we trained bees to two such artificial feeders by capturing outgoing bees at the entrance of the colony and transferring them to a feeder. The initial location of the feeders was directly in front of the hive and they were moved a few meters once enough bees were visiting regularly. The feeders reached their final positions on 2019 August 21: Each was approximately 210 m from the hive with feeder 1 east-northeast of the hive (52.457711, 13.299308) and feeder 2 southwest from the hive (52.455709, 13.294238). After each day of recording, we transferred the video material to a Network Attached Storage. We processed the full video material after the season with the same software that we used for the in-hive recordings. The sugar concentration was always the same for both feeders but varied between 0.5:1 sugar:water and 1:1 sugar:water before the 2019 August 28 after which it was held constant at 3:2 sugar:water. We added a drop of anis solution to the sugar solution before splitting it for the two feeders to ensure that both feeders had the same odor. On specific dates (2019 August 30, 2019 September 4, 2019 September 12, 2019 September 15) we performed experiments with a third feeder that was either close to feeder 1 or to feeder 2, where the original feeders contained only water. On these days, we alternatingly carried over bees caught at the hive exit or at the farther feeder to feeder 3 to trigger dances to a new forage site.

#### Berlin 2021

Between 2021 August 27 and 2021 September 15, we conducted experiments with two feeders at varying locations around the hive, so the recorded data contain both dances to natural forage sites and to our feeders. Since the bees were not marked individually, we used classical feeders without cameras. We did not scent the sugar solution this season.

### Data processing

#### Rotation correction

As some tags were not glued onto the bees perfectly aligned with their body orientation, we postprocessed the decoded orientations to correct for offsets. We had observed that bees that move quickly tend to move forward rather than sideways or backwards. So we used the average difference between movement direction and decoded tag orientation of a bee over her life to calculate an offset value for each bee that was then used to correct the tag orientation. For that, we collected all detections with at least 0.1 detection confidence from all tracks of each bee over their life. For each track with at least six detections (ca. 1 s), we calculated the movement direction as the angle between the *x* and *y* position of successive detections. For each of these tracks, we calculated the circular mean of the differences between body orientation and movement direction. We disregarded all short tracks and all tracks with a high standard deviation of the mean differences and kept only the higher resp. lower 50%. We generated a histogram with 10${}^{\circ }$ bins and disregarded all values that did not fall into the mode of the histogram. The final orientation correction was then the median of all remaining values.

#### Interpolation

For single timesteps where we did not have detections for a bee available, we linearly interpolated the position and orientation between adjacent timesteps.

### Area covered by dance floor and dances

To calculate the fraction of the comb that is used as the overall dance floor, we used all days for each side of the hive that had more than the median number of detected waggle phases on that side to disregard days where no proper dance floor could emerge due to low activity. We subdivided the available comb (without the wooden frame) into a grid of $1\;{\mathrm{cm}}^{2}$ cells and sorted all waggle phases into their respective cells, creating a 2D histogram of counts. We counted the number of cells that covered 95% of the total number of waggle phases and divided that by the total number of cells on the comb to get the fraction of the comb covered by the dance floor. To estimate the area covered by the dances to the individual feeders, we took a similar approach. We disregarded all days for each side where the number of dances towards that feeder was lower than the median number of dances per day to that feeder and side. Instead of the individual waggle phases (which are not available for every dance), we used the location of all detections of each individual dancer during their dance to create the histogram.

### Waggle drift motion model

We used a simple step-based motion model to simulate the locations of successive waggle phases. We simulated 2,000 dances each consisting of 20 waggle phases. For each dance, we first sampled an initial start location from a Gaussian distribution as $\left(x,y)∼(N(\mu =80,\sigma^{2}=20),N(\mu =160,\sigma^{2}=20)\right)$ and a random waggle direction from a uniform distribution α∼U(0,2π). Every waggle phase of a dance then constituted one simulation step, i.e. we updated the initial position of each dance 20 times and saved the resulting 20 locations as our waggle phase locations. For each of these steps, we sampled a forward offset $o_{\;f}∼N(\mu =0.80834,\sigma^{2}=7.30946)$ that was added to the position in the waggle direction α and a sideways offset $o_{s}∼N(\mu =0,\sigma^{2}=8.15597)$ that was added to the location orthogonal to α. If the new location was outside of the comb area (400×240), the sampling was repeated until the new spot was inside the comb.

### Waggle phase detection

A second pair of cameras observed the hive from both sides at a lower spatial resolution (ca. 40 pixels per bee length) but a higher frame rate (60 Hz). This camera stream was evaluated in real-time to detect image regions that showed periodic brightness changes of around 13 Hz, i.e. regions with potential waggle dances. These detections were then further processed to filter out nondances, decode the waggle orientation and duration, and map these detections to the field. Since the original publication of the waggle dance detector (WDD) (7), we continued to improve the software which is available on GitHub.^2^ For the data recorded in 2019, we used the original decoding of the waggle angle, but filtered the recorded waggle phases with a semisupervised machine learning approach: we trained a contrastive-predictive-coding (CPC) model (36) on the short video sequences that were saved by our software for each detection. Then, we annotated a number of waggle phases as either real or false detections and used a simple logistic regression model based on the CPC embeddings to predict whether a waggle was a real detection. We retrieved the waggle phases with the lowest prediction confidence and annotated them as well to improve the classification of difficult samples. For processing the 2021 data, we further improved the resilience of the first stage of the WDD by adding an additional background subtraction step that reduces the impact of lights flickering in the video. Also, in order to improve the accuracy of the final detections, we implemented a convolutional neural network to sort and decode the detections of the first stage of the WDD. From the data we recorded from the observation hive Berlin 2021, we randomly sampled 1,009 detections for which we looked at the recorded sequences of crops from the full video stream and manually sorted them into the classes *waggle phase* (N=246), *shaking signal* (N=293), *ventilating* (N=97), and *other* (N=373). We also annotated the body orientation of the bee in the center of the crop that triggered the detection. We chose a neural network architecture consisting of seven stacked 3D convolutional layers using the Mish activation function (37) with 3D BatchNorm (38) before each activation. Before the activation of the final hidden layer, we use a Gated Linear Unit (39) and we use feature-map based SpatialDropout (40) after the 5th convolutional layer as a regularizer. The model has 121,431 parameters. The network takes a 40 frames sequence of 32×32 crops at half the original resolution. During training, this sequence was randomly selected as a subset of the available frames (usually around 200) per sample, which was augmented with multiple standard transformations (random affine transformation, random noise, random brightness and contrast changes). We trained the network on 90% of the ground truth data with the Madgrad optimizer (41) for 48 epochs with each epoch consisting of 1,000 iterations over the training set. In each epoch, we used the learning rate scheduler introduced in Ref. (42). On the test set, the trained model achieved an average one-versus-all area under the receiver operating characteristics curve (ROC AUC score) of 97.8%, an average weighted (by sample count) harmonic mean of precision and recall (F1 score) of 91%, and for the *waggle phase* class a recall of 83.3% and a precision of 78.9% (see Ref. (43) for a review of the different classification metrics). The cosine similarity of the body orientation for the *waggle phase* was 0.92. The architecture and code to train the network are available on GitHub.^3^

#### Dance clustering

In order to further reduce noise, we clustered the detected waggle phases into dances by considering all waggle phase detections that were less than 3 cm and 5 s apart to belong to one dance. We disregarded all clustered dances shorter than three waggle phases. For the remaining ones, we calculated an agreement on the waggle angle using random sample consensus (RANSAC) (44) with at least 60% required inliers. We disregarded waggle phases for which no RANSAC agreement could be found.

### Dance and dance-following classifier

#### Ground truth data

To train the neural network that classifies dancing and dance-following, we needed annotated ground truth data. We decided to individually annotate the start and end timestamps of dance-following behavior and the start and end timestamps of individual waggle phases for dancing bees. To generate data for subsequent annotation, we randomly sampled 5-min intervals from the recording period (2019 August 1 to 2019 September 23). For these intervals, we queried our WDD detections for all waggle phases that fell into each interval. We generated a video from our HD data for each of those detections, combining detections that were less than 30 s apart into one video. The videos had an additional margin of 10 s before the first and after the last detection. In addition to the video data, we queried our positional data to retrieve all the bees that were contained in the video and saved them as tracks. We used the BioTracker (45) to load the videos and the tracking data and manually annotate each waggle and following behavior in each of the videos. In total, we annotated 176 videos, containing 3,278,121 detections from 28,873 tracks by 4,389 unique bee IDs, labeling a total of 1,107 distinct waggle phases and 279 follower events. We then considered all detections in all frames of the videos as distinct samples, with detections that were not annotated as either a waggle phase or a following event being labeled as negative samples. This yielded 3,250,375 negative samples, 6,773 waggle phase samples, 20,817 dance-following samples. We assigned all samples from each hour to a distinct sample group, which we subsequently used to split the data for cross-validation to not have strongly correlated samples in the training and testing set. We also used additional unsupervised data for the training. We sampled 100,000 random waggle phases from our waggle dance recognition (WDD) between the dates of 2019 August 20 and 2019 September 20 and subsequently retrieved all individual IDs of bees that had at least three detections with at least a confidence of 0.1 in a square of 1-by-1 cm in a 2 s window around the WDD detection. That way we generated an additional 314,274 unsupervised samples.

#### Data preprocessing and features

For each of the samples, we queried our detection database for the position and orientation of all detections with the same ID in a time window of 12 frames (2 s) for the embedding model and 54 frames (9 s) of the trajectory model, respectively. If detections were missing for single frames (e.g. due to obstruction of the bee), these frames were filled by linear interpolation. If more than 50% of frames were missing, the samples were dropped. As an additional feature, a binary mask was generated that was 0 for interpolated frames and 1 otherwise. The *x*, *y* coordinates, and orientation were transformed into ego-motion perspective: for each frame, the instantaneous velocity was calculated and median filtered with a kernel size of 3. The sine and cosine of the motion direction relative to the individual’s orientation were calculated. The change in orientation of the bee was given by the difference of the sine and cosine of its orientation from the last frame. The final features were then: velocity, cos(movement direction), sin(movement direction), delta cos(orientation), delta sin(orientation), mask. All features were clipped at the 5th and 95th percentiles and then z-transformed following standard practices. For the cross-validation, the percentiles and centering parameters were extracted on the training set of the embedding dataset and then applied to the other datasets.

#### Models

The model consists of two main parts: an embedding model that calculates an embedding vector from a short sequence of a trajectory, and another small model that takes a series of embeddings and classifies each timestep into one of the classes *other*, *dancing*, *following*. Both models are fully convolutional with stacked 1D-convolutions, using exponential linear units (ELU) as their hidden layers’ activation functions (46). The embedding network is trained with two losses: a weighted cross-entropy loss with the respective class labels for the labeled samples, and the InfoNCE loss used with the unlabeled and labeled samples in a similar way as in Ref. (36). For each sample of the training data, two sequences of trajectories are extracted: one 12 frames sequence centered around the sampling timestamp $t_{1}$ and the subsequent 12 frames $t_{2}$. The respective embeddings $e_{1}$ and $e_{2}$ of both sequences are calculated and with one linear layer a predicted embedding $p(e_{1})$ is calculated. For a batch of size *b*, we calculate the dot products of all $p(e_{1})$ and all $e_{2}$ as a matrix of size b×b. The mean row-wise cross entropy constitutes the final unsupervised loss. All $e_{1}$ of all labeled samples in each batch are passed through a small three-layer fully connected perceptron using ELU activation and BatchNorm (38) to predict the likelihoods for the three classes and calculate the samples’ mean cross-entropy loss. In addition to the two losses, the embeddings are L1-regularized. The full code to train the models is on GitHub.^4^

#### Training

The models were trained with the Madgrad optimizer (41) for around 180,000 batches with batch size 1,024 for the embedding submodel and 36,000 batches with batch size 512 for the full (embedding + trajectory) model. The learning rate followed a cosine-annealing schedule with regular restarts (47). When evaluated on the middle timestep of each test-set sequence, the trained model reached a ROC AUC score of 98.58%, an accuracy of 87.85%, an F1 score of 67.17% and a precision for the *dancing* class of 53.92%. We then further improved the precision with postprocessing.

#### Postprocessing

To apply the model to all available data for a whole day, we first selected all bees that were alive on that given day (determination of alive status as in Ref. (22)). We then stepped through the frames for each side of the hive in steps of 30 frames (ca. 5 s) and queried the trajectory of each bee with a length of 108 frames (ca. 18 s) centered on the selected frame. We then calculated the features for the trajectories as described above and applied the model. We then applied a median filter with a kernel size of 5 to the softmax output. To determine the output class, we then used the argmax of the prediction for each timestep. To further filter out incorrect detections, we then considered all adjacent detections of the “dance” class that are at least 4 s long (and contain gaps of up to 3 s) as “dance events.” For each frame of each dance event, we then queried all our detections from our database that were at maximum 14 mm away from the dancing bee and had a nonnegative dot product of their orientation vector and the vector towards the dancer (i.e. that approximately faced the dancer). We then labeled all these potential followers that we saw for at least 1 s as either *attendee* or *follower*. Bees that were assigned the “follower” label from our model at that point in time were subsequently treated as followers; all others are *attendees*. We then disregarded very short attention or following events that lasted less than one second (subsequent events with gaps of less than 3 s were merged into one). Since we did this assignment of the attendee or follower state for each detected dance, we also had the mapping between dancer and follower. To remove remaining false positive detections during our feeder experiments, we only considered dances where we had seen the individual bee at a feeder in the last five minutes prior to the dance, which we also used to assign that dance to a feeder. We disregarded one data point of a bee that was seen at both feeders in that interval, but otherwise did not further remove data points of bees that might have switched between feeders during the experiment. We manually inspected a sample of 55 dance detections and confirmed they were indeed all dances.

## Supplementary Material

pgad275_Supplementary_Data

## Data Availability

The data and corresponding code are available online as Open Access (48).

## Footnotes

1
https://github.com/BioroboticsLab/bb_raspicam

2
https://github.com/BioroboticsLab/bb_wdd2

3
https://github.com/BioroboticsLab/bb_wdd_filter

4
https://github.com/BioroboticsLab/bb_dance_networks
