# Supplementary material for: Machine learning reveals the waggle drift’s role in the honey bee dance communication system
Source: PNAS Nexus. 2023 Aug 25;2(9):pgad275. doi: 10.1093/pnasnexus/pgad275 (PMC10516631; doi:10.1093/pnasnexus/pgad275)
Supplement: pgad275_Supplementary_Data [file pgad275_supplementary_data.pdf]

Machine learning reveals the waggle drift's role in the honey bee dance communication system.

David M. Dormagen[a,*], Benjamin Wild[a], Fernando Wario[b], Tim Landgraf[a]
[a]Department of Mathematics and Computer Science, Freie Universität Berlin, Berlin, Germany
[b]Department of Electronics, Universidad de Guadalajara, Jalisco, Mexico
[*]To whom correspondence should be addressed: david.dormagen@fu-berlin.de

Supplementary Material

List of Figures

Fig. S1 Location of waggle phases on the comb 2
Fig. S2 Quiver plot of observed waggle phases 3
Fig. S3 Location of dances advertising the two feeders 4
Fig. S4 Simulated waggle phases 6
Fig. S5 Simulated waggle phases - null model 7
Fig. S6 Histogram of forward drift in manually annotated data 8
Fig. S7 Effect on dance-following interactions - distance to the followed dance 9
Fig. S8 Effect on dance-following interactions - distance to the dance advertising the same/other feeder 10

List of Tables

Table S1 Overview of detection statistics per dataset and comb side 5

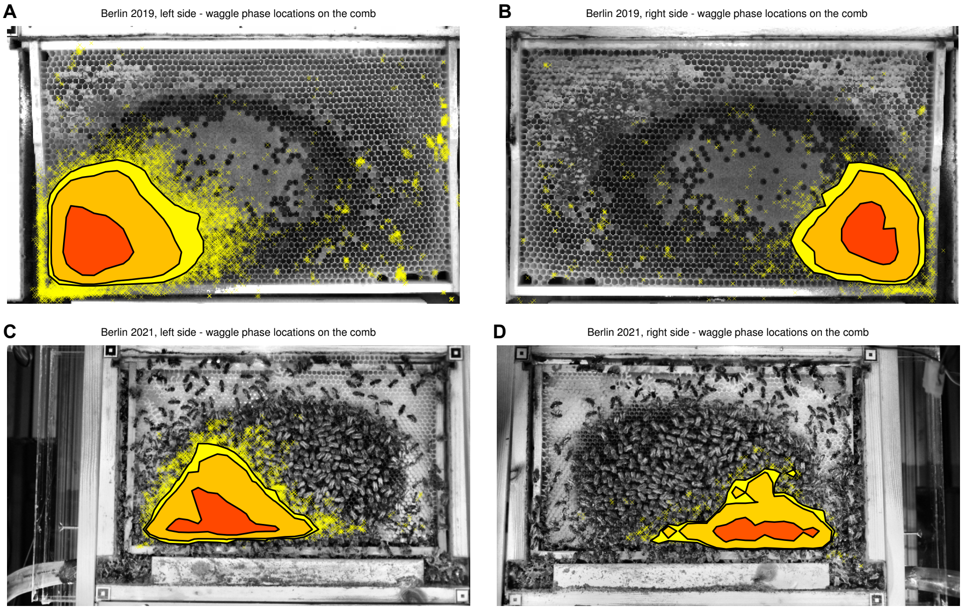

Fig. S1. Location of waggle phases on the comb. Each automatically detected waggle phase is displayed on top of an image from our recording (background subtracted images are used for 2019). The waggle phases cluster in a region close to the exit (the exit tube is visible in the images from 2021). As the scatterplots are too dense in the central regions, contour plots indicate 50%, 90%, 95% of the density. A: Berlin 2019, left side (N=84,628 from 32 days) B: Berlin 2019, right side (N=14,404 from 31 days) C: Berlin 2021, left side (N=36,367 from 47 days) D: Berlin 2021, right side (N=6,390 from 33 days)

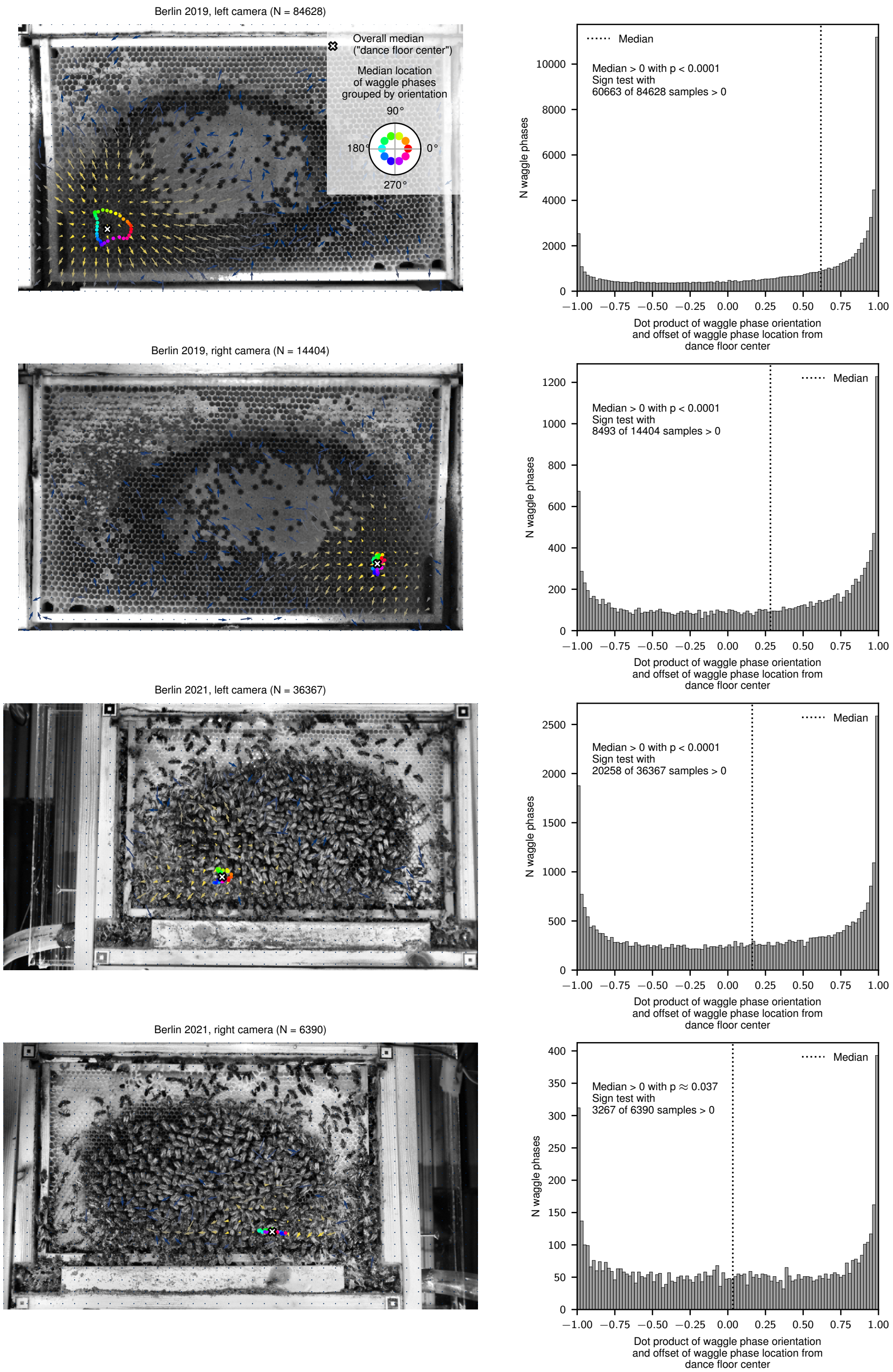

Fig. S2. Quiver plot of observed waggle phases. A mean orientation is calculated for each cell of a 1 cm^2 grid. For each 5° circle segment, the median position of the subset of waggle phases with an orientation that falls into that segment is calculated and displayed as a colored dot on the comb. In its circular colormap, red corresponds to 0° resp. 360° and blue hues correspond to around 180°. The right column shows histograms of the dot product of the orientation of each waggle phase and its normalized offset from the dance floor center. The dot product ranges from -1 to 1 and is close to 1 if the waggle phase is offset from the center in the same direction as indicated by its orientation. In all datasets we see that the median of this dot product is significantly larger than 0 (for the p-values and test statistics refer to the individual subplots). In the dataset from the right camera of 2021, the relationship is the weakest. This might be due to a combination of a lower number of samples and the waggle phases only advertising a smaller subset of all possible waggle orientations, which shifts the overall median position (the dance floor center) away from where it would be if we would observe a more even number of dances for each possible orientation.

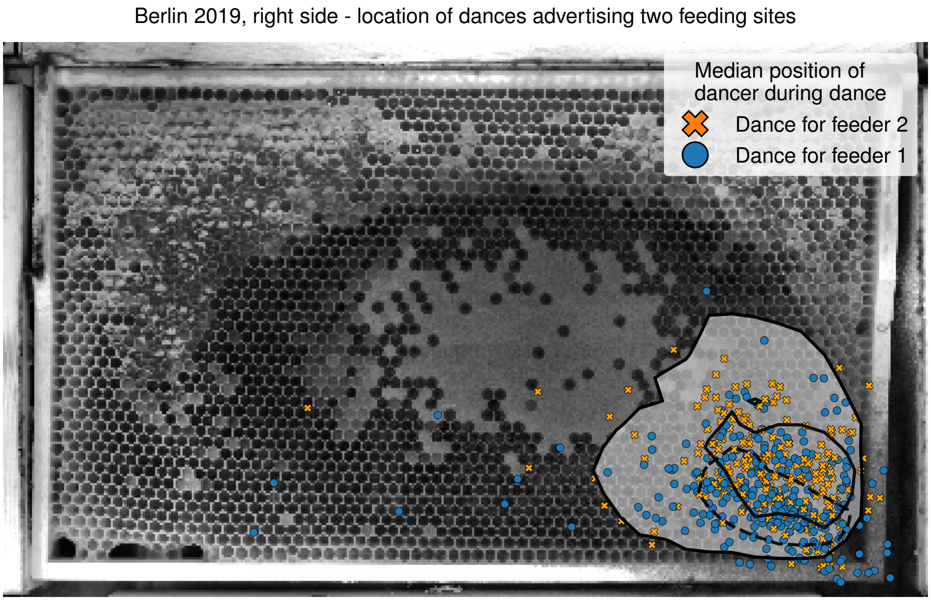

Fig. S3. Location of dances advertising the two feeders. This figure shows the dance locations recorded in Berlin 2019 on the right side of the comb. The scatterplot shows the median position of each automatically recognized dance from bees returning from feeder 1 (blue) or feeder 2 (orange) (N=496). The two overlapping contours give the 50% density area for feeder 1 (dashed) and feeder 2 (solid). The large area shows the 95% data interval of all waggle phases again for reference.

	Berlin 2019 – left side	Berlin 2019 – right side	Berlin 2021 – left side	Berlin 2021 – right side
# individual dances	1497	496		
# dances to F1	840	291		
# dances to F2	657	205		
# days with at least one dance to F1	15	14		
# days with at least one dance to F2	15	12		
Median dances per day to F1	30	18		
Median dances per day to F2	24	14.5		
% of comb used as dancefloor to F1	9.46% ± 2.41%	7.89% ± 1.99%		
% of comb used as dancefloor to F2	10.20% ± 3.43%	9.50% ± 2.61%		
# waggle phases detected	84628	14404	36367	6390
# days with at least one waggle phase	32	31	47	33
Median waggle phases per day	1308.5	236	346	93
% of comb used as dancefloor (estimated from days with more than the median waggle phases)	11.64% ± 1.86%	7.10% ± 1.80%	8.74% ± 3.39%	4.92% ± 1.54%

Table S1. Overview of detection statistics per dataset and comb side

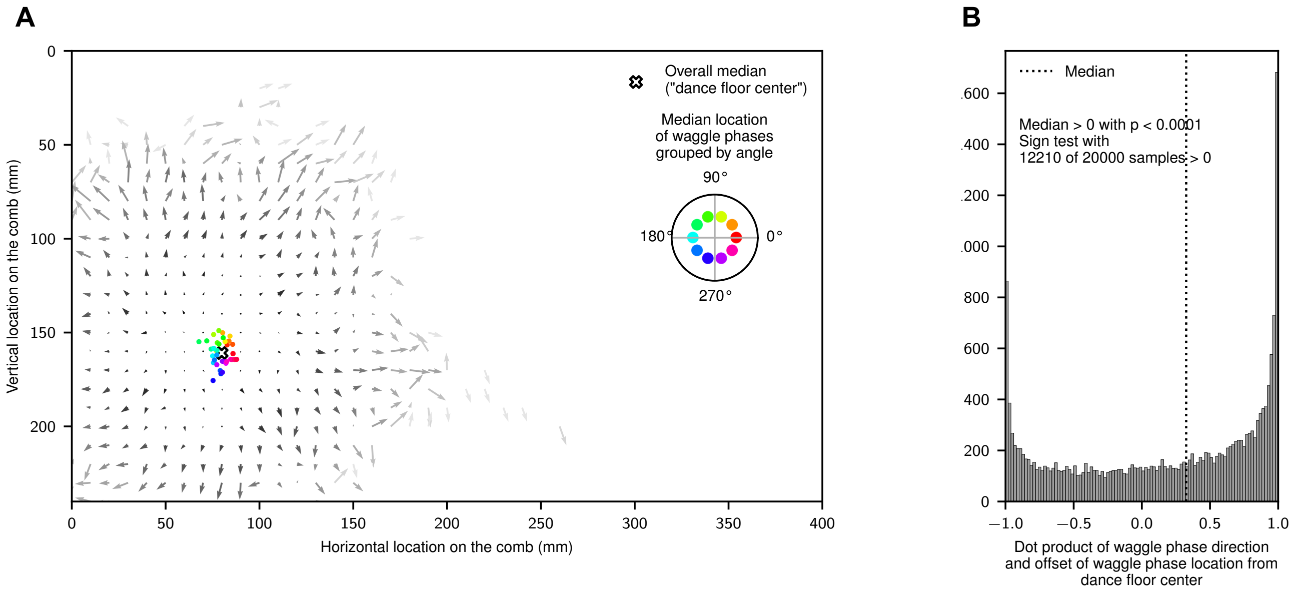

Fig. S4. Simulated waggle phases. A: Quiver plot of simulated waggle phases. A total of 40,000 waggle phases were simulated for 2,000 dances with 20 waggle phases each. Each arrow indicates the mean direction for a 1 cm^2 cell. The simple motion model based solely on the drift between subsequent waggle phases results in a similar pattern as we observe in our experiments. B: Histogram of the dot product of the orientation of each waggle phase and its normalized offset from the dance floor center.

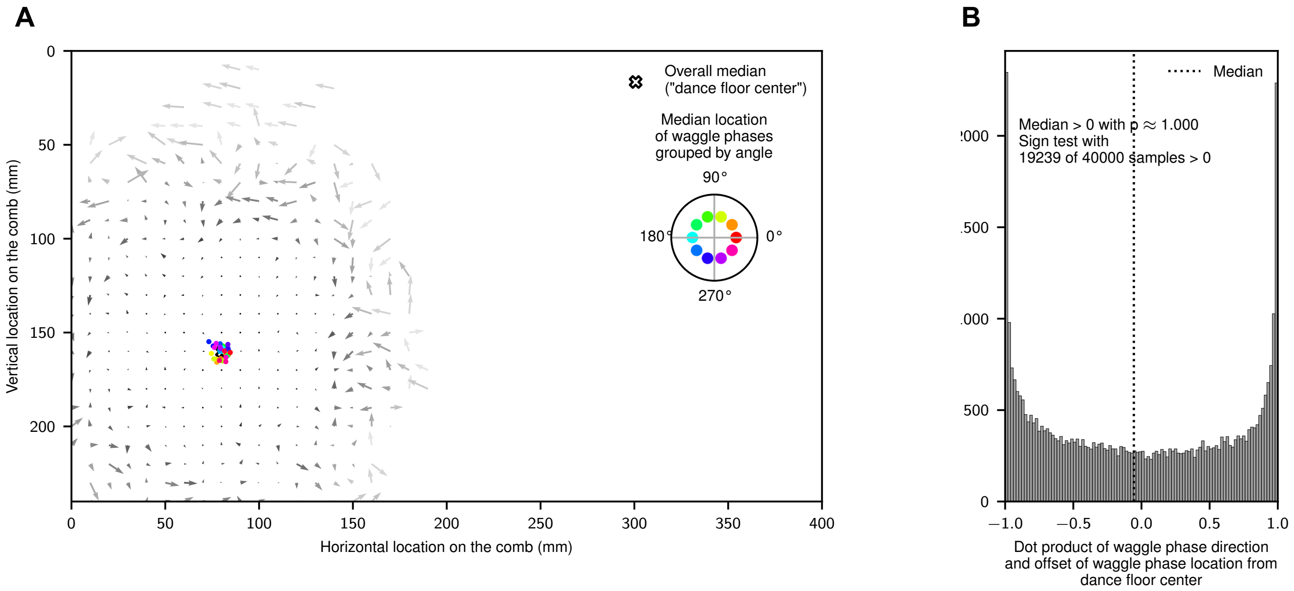

Fig. S5. Simulated waggle phases - null model. Results from our waggle drift motion model with the mean forward drift set to 0 mm instead of 0.81 mm. A: Quiver plot of simulated waggle phases. A total of 40,000 waggle phases were simulated for 2,000 dances with 20 waggle phases each. Each arrow indicates the mean direction for a 1 cm^2 cell. B: Histogram of the dot product of the orientation of each waggle phase and its normalized offset from the dance floor center. The distribution with its peaks at -1 and 1 and median of 0 has an identical shape to a distribution of the cosine of uniformly distributed angles.

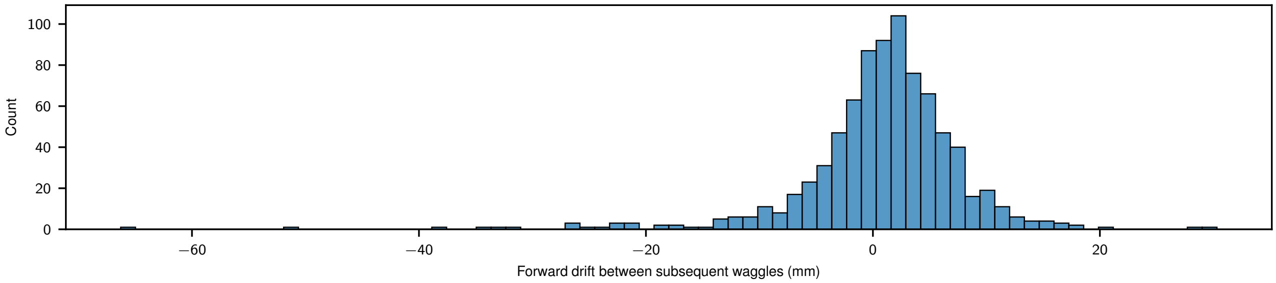

Fig. S6. Histogram of forward drift in manually annotated data. Looking at all pairs of subsequent waggle phases (N=820) in the manually annotated data, we find a positive forward drift, i.e. an offset between subsequent waggle phases in the direction of the waggle phase, albeit with a high variance.

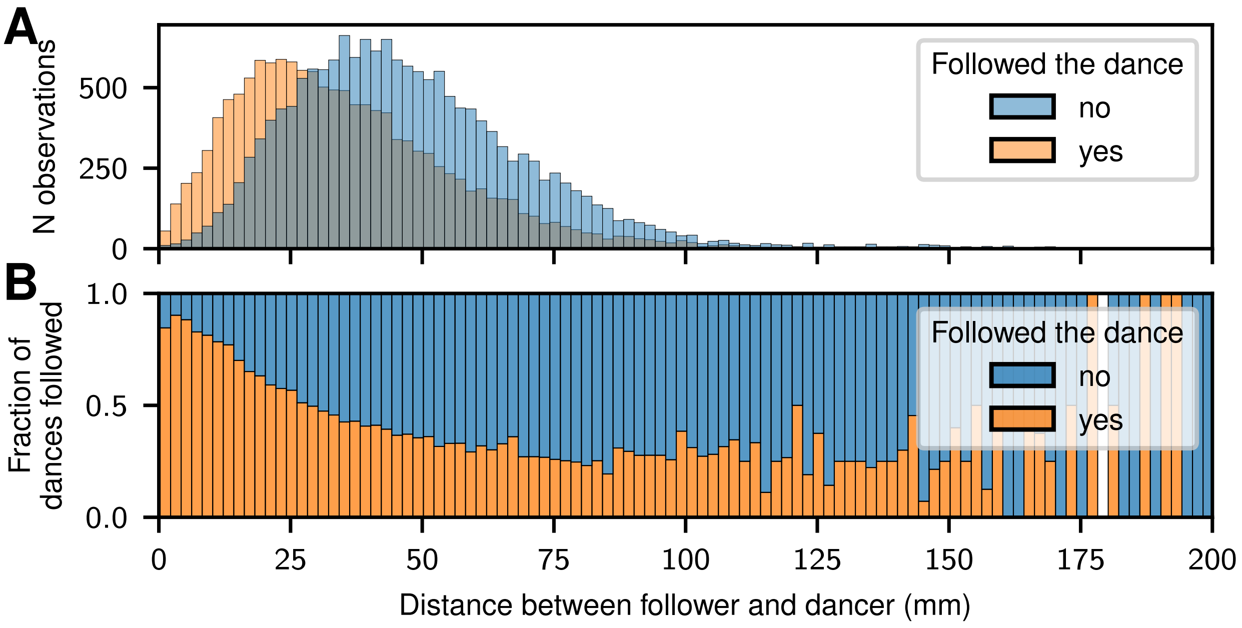

Fig. S7. Effect on dance-following interactions - distance to the followed dance. A: Histogram of the number of dances over distance to the follower bee in situations where after an initial dance-following interaction at least two dances to the different feeders were available. The dance that is eventually followed is on average closer. B: Same data as in panel A, normalized per distance bin. Note that this still only includes situations where two subsequent dances were available after one initial dance-following interaction and should not be interpreted as a general likelihood of a bee following a dance based on distance.

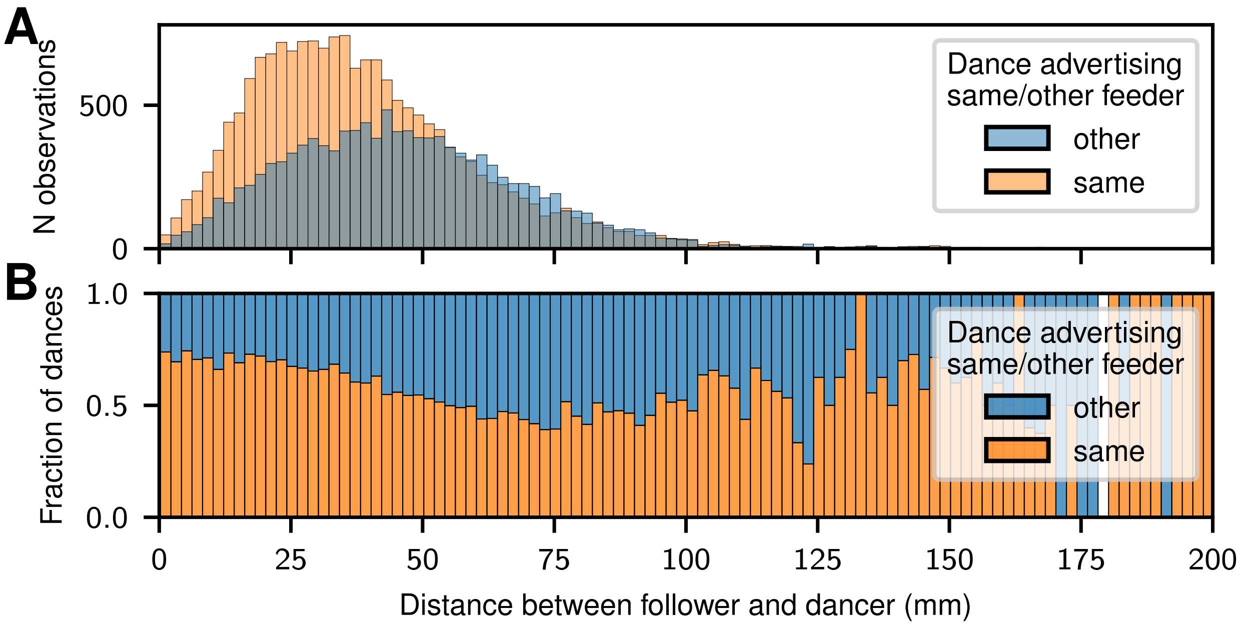

Fig. S8. Effect on dance-following interactions - distance to the dance advertising the same/other feeder. A: Histogram of the number of dances over distance to the follower bee in situations where after an initial dance-following interaction at least two dances to the different feeders were available. The dance that advertises the same food site as the initial dance is on average closer. B: Same data as in panel A, normalized per distance bin.
